# Increased *c-myc* and *mi*R-33 Expression in Expanded Hematopoietic Stem Cells Cultured on Adipose Stem Cells Feeder Layer

**Published:** 2017-11-01

**Authors:** T. Foroutan

**Affiliations:** Department of Animal Biology, Faculty of Biological Sciences, Kharazmi University, Tehran, Iran

**Keywords:** Fetal blood, Cord blood stem cell transplantation, MicroRNAs, Mesenchymal stromal cells, Genes, p53, Genes, myc, MIRN33 microRNA, human [Supplementary Concept]

## Abstract

**Background:**

Umbilical cord blood has been used for transplantation in regenerative medicine of hematological disorders. MicroRNAs are important regulators of gene expression that control both physiological and pathological processes such as development and cancer. Some studies have shown that *miR-33, p53* and *c-myc* have critical roles in control of self-renewal cells.

**Objective:**

To understand the effect of adipose-derived mesenchymal stem cells (ADSCs), as a feeder layer, on expansion of HSCs, the expression of *p53* and *miR-33a* were evaluated.

**Methods:**

Isolated human ADSCs in passage 3 were cultured as a feeder layer. *Ex vivo* cultures of cord blood CD34+ cells were performed in three culture conditions for 7 days: cytokines with ADSCs feeder layer, cytokines without ADSCs feeder layer, and co-culture with ADSCs without cytokine. Expression of genes *p53, c-myc* and *miR-33* were analyzed by real-time PCR.

**Results:**

The expression of *p53* was significantly down-regulated in HSCs directly cultured on ADSCs feeder layer compared to that cultured without feeder layer. The expression of *miR-33a* was significantly up-regulated in HSCs directly cultured on feeder layer compare to that cultured without feeder layer.

**Conclusion:**

Defining the role of ADSCs in controlling the HSC self-renewal through *miR-33*, *p53* and *c-myc* may lead to the treatment and prevention of hematopoietic disorders.

## INTRODUCTION

Umbilical cord blood (UCB) has been used for transplantation in regenerative medicine of hematological disorders. Attempts to improve hematopoietic reconstitution and engraftment potential of *ex vivo* expanded hematopoietic stem and progenitor cells have largely been unsuccessful due to the inability to generate sufficient stem cell numbers and to excessive differentiation of the starting cell population [1]. *In vitro* studies showed that control of hematopoietic stem cells (HSCs) self-renewal in culture is difficult.

Hematopoietic cytokines have failed to support reliable amplification of HSCs in culture; it seems additional factors to be required. Recently, factors such as feeder layer have been reported to affect HSC expansion. Mesenchymal stem cells, as a feeder layer, could prevent apoptosis of expanded hematopoietic stem cells derived from cord blood [2]. Adipose stem cells (ASCs) show properties similar to that observed in bone marrow mesenchymal stem cells. Due to easy accessibility, human adipose-derived mesenchymal stem cells (hADSC) are an attractive source for regenerative medicine [3]. ASCs have been shown to be immunoprivileged, prevent severe graft-versus-host disease *in vitro* and *in vivo* and to be genetically stable in long-term culture. They have also proven applicability in other functions, such as providing hematopoietic support and gene transfer [4]. ADSCs exhibit high intrinsic expression of self-renewal supporting factors compared to bone marrow mesenchymal stem cells [5]. Since ADSCs produced various factors to support stem cells maintenance and cell growth, here we used them as a feeder layer for expansion of HSCs.

MicroRNAs are important regulators of gene expression that control both pathophysiological processes such as development and cancer [5]. MicroRNAs are short noncoding RNAs, usually 18–25 nucleotides in length, which repress translation and cleave mRNA by base pairing to the 3’ untranslated region of the target genes [7]. It has been demonstrated that miRNAs play important roles in developmental biology, cellular differentiation programs and oncogenesis [8]. They regulate various cellular processes of tumor, including cell proliferation, differentiation, progression, apoptosis and invasion [9, 10]. Alterations in the miRNA expression have emerged as in important mechanism for the development and progression of cancers [11, 12]. Various number of miRNAs were studied in HSCs. The role of *miR-33* in regulating cell proliferation and cell cycle progression is the subject of many investigations. It has been shown that *miR-33* family members modulate the expression of genes involved in cell cycle regulation and cell proliferation [13]. Various functions have been defined for *miR-33* such as reduction of cell proliferation and cell cycle progression and impairing the *p53* tumor suppressor gene function.

The function of *miR-33* is associated with genes such as *p53* and *c-myc*. One of the cell cycle inhibitor genes is tumor suppressor gene *p53*. Previously, it has been reported that *miR-33* targets *p53* [13]; *p53* activates the transcription of genes that induce cell cycle arrest, apoptosis and senescence in response to several stress conditions including DNA damage [13]. In addition to *p53* gene, *c-myc* gene is involved in cell proliferation by accelerating cells through G1 and S phases of the cell cycle and abrogating cell cycle checkpoints [13]. Delgado and colleagues in 2010 reported that *c-myc* is important for correct balance between self renewal and differentiation of HSCs [14]. *c-myc* as an effector molecule of Notch signaling pathway and HOXB4 can increase the HSCs proliferation [15]. On the other hand, it was reported that there is a negative relationship between *miR-33* and *c-myc* so that over-expression of *c-myc* impaired miR-33b-induced inhibition of proliferation and invasion in osteosarcoma cells [16, 17]. In this study with regard to the roles for *p53*, *c-myc* and *miR-33* in self-renewal of HSCs, the effects of ADSCs, as a feeder layer on HSCs cultures, was investigated.

## MATERIALS AND METHODS

Cell Culture

Human subcutaneous adipose tissue samples were obtained from donors during abdominoplasty. The tissue samples were processed using a modified procedure by Zuk, *et al* [18], with 0.075% collagenase II (Sigma-Aldrich, St. Louis, MO, USA) for 30 min. The samples were then centrifuged at 150×g for 5 min. The pellet was washed three times in phosphate buffered saline (PBS). The cells seeded in 10^5^ cells/dish and cultured in Dulbecco’s Modified Eagle’s medium (DMEM), 10% fetal bovine serum (FBS), and 100 U/mL penicillin/streptomycin. Human HSCs were taken from Royan institute.

Proliferative and Phenotypic Analysis

Surface markers of ASCs were analyzed by flow cytometry, and monoclonal antibodies were used against for CD73, CD90 and CD105 markers. To differentiate into adipocytes, ASCs from passage four and a medium containing 10 nM dexamethasone, 5 mM NaCl, 10 mM IBMX, and indomethacin was used. To differentiate into osteoblast cells a medium of DMEM, high glucose with 10% FBS and 10 nM dexamethasone, 35 mg/mL of ascorbic acid, and 1 mM β-glycerophosphate was used. Cells were incubated in 5% CO_2_, at 37 °C for 21 days. To demonstrate the differentiation into adipocytes and osteoblasts, alizarin red and oil red were used.

CD34+ cells Isolated from UCB

After obtaining written informed consent, mononuclear cells with Ficole (Sigma, 1.077±0.001 kg/L) were separated. These cells were then incubated with anti-CD34 antibody labeled with nanoparticles of Fe (America Milton Biotech); CD34+ cells were separated by column MACs (America Milton Biotech). Anti-CD34 and anti-CD38 were used to confirm the presence of CD34 marker for cells isolated from UCB.

Culture of CD34+ cells

**Figure 1:**
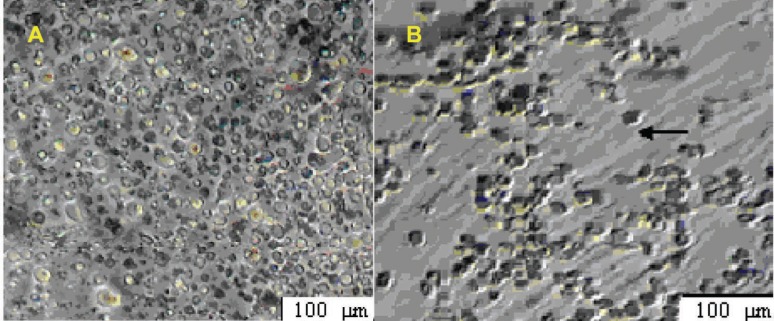
HSCs were cultured on ASCs feeder layer (A) after 2 days, (B) after 7 days

After preparation of the feeder layer with mitomycin c, CD34+ cells were cultured for 7 days with 100 ng/mL cytokines such as stem cell factor( SCF), thrombopoietin (TPO), fetal liver tyrosine kinase 3 ligand (Flt-3L), and stem span medium as follows: (1) only in the presence of the mentioned cytokines, (2) directly in contact with ASCs feeder layer, and (3) indirectly cultured on ASCs feeder layer (cultured on Thincert plate with 0.4-µm pore size) (Fig 1).

In addition to this group, another group of cells without culture were used as control and were analyzed immediately after their extraction.

MTT Assay

The MTT (3-[4, 5-dimethylthiazol-2-yl]-2, 5-diphenyltetrazolium bromide) assay was used to assess cell viability of all studied groups. This assay measures the amount or ratio of cell proliferation. It is a colorimetric method depending on the reduction of the tetrazolium salt, MTT, to form blue formazan crystals. After completion of the incubation, the overlying culture medium was removed. After the addition of MTT, cells were incubated for four hours in an incubator containing CO_2_ at 37 °C.

Isopropanol acid was added and the optical density (OD) of the obtained solution was read at 630 nm, as the reference wavelength, and at 570 nm, as the measurement wavelength, using an ELISA reader. One-way ANOVA was used for data analysis.

Apoptosis Analysis by Annexin V and Propidium Iodide

Apoptosis kit (Bioscience, USA) was used for apoptosis analysis. At 14^th^ day of culture, 10^4^ cells after being resuspended in 1× binding buffer were treated with fluorochrome-conjugated Annexin V for 10 min and then washed and resuspended in 1× binding buffer. Next, propidium iodide solution was added and fluorescence of the stained cells was analyzed by flow cytometry.

RT-PCR

After the RNA extracted from cells by Trizol, for reverse transcription of mRNA, cDNA was synthesized from 2 µg of total RNA. For cDNA synthesized Random Hexamer and Oligo dt primers were used. GAPDH and U48 were used as loading controls for quantitation of mRNA and miRNAs.

The sequences of GAPDH, *p53* and *c-myc* are as follows:

p53

Forward:

 5’ TCCTCAGCATCTTATCCGAGTG 3’

Reverse:

5’ AGGACAGGCACAAACACGCACC 3’

c-myc

Forward:

 5’ CAAGAGGCGAACACACACAACGTCT 3’

Reverse: 5’ AACTGTTCTCGTCGTTTCCGCAA 3’

GAPDH

Forward:

 5’ ATGGGGAAGGTGAAGGTCG 3’

Reverse: 

 5’ GGGGTCATTGATGGCAACAATA 3’

miR-33a

Forward: 

5’ GTGCATTGTAGTTGCATTGCA 3’

Reverse:

5’ TGACCCCAGGTAACTCTGAGTG 3’

Real-time PCR

Real-time PCR was performed with an Evagreen and data were analyzed using the formula 2-ΔΔct.

Statistical Analysis

Data are presented as mean±SD. Two-way ANOVA and Duncan tests were used. A p value <0.05 was considered statistically significant.

## RESULTS

**Figure 2:**
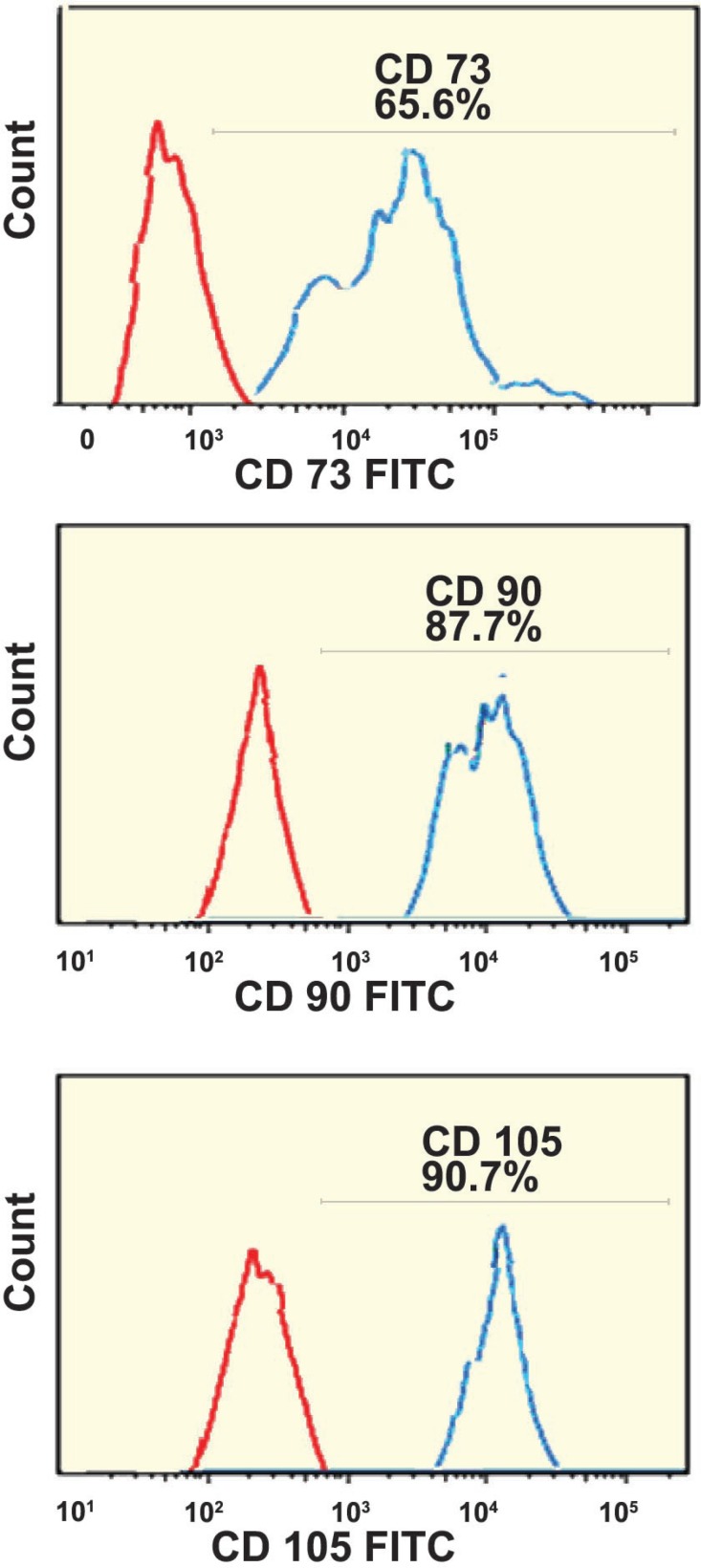
Analysis of ADSCs markers with flow cytometry. 87.7% of ADSCs were positive for CD90, 65.6% of the cells were positive for CD73, and, 90.7% of ADSCs were positive for CD105

Surface antigen markers of ASCs were analyzed by flow cytometry. Our results showed that ASCs were positive for mesenchymal stem cells markers such as CD105, CD90, and CD73 and negative for HSC markers such as CD34, and CD45 (Fig 2).

**Figure 3:**
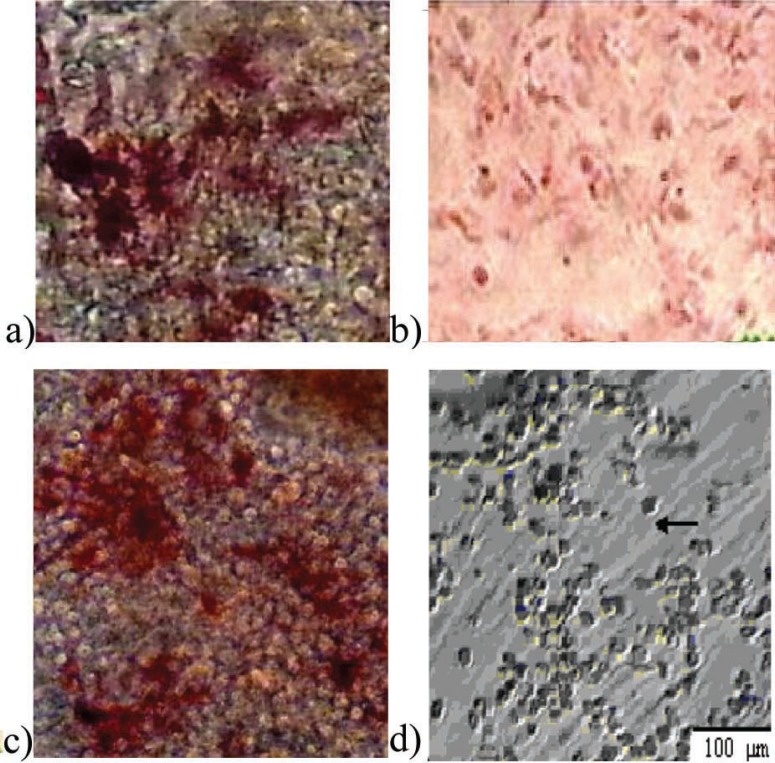
Osteogenic differentiation of adipose-derived mesenchymal stem cells (×200). (a) Positive reaction in osteoblastic differentiated cells with alizarin red staining; (b) undifferentiated cells; (c) osteoblastic differentiated cells with increased alkaline phosphatase activity; (d) undifferentiated cells

**Figure 4:**
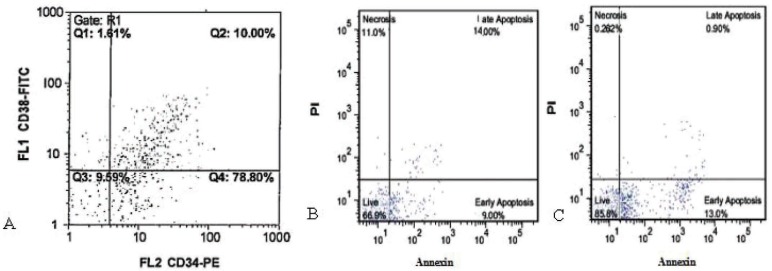
Flow cytometric analysis of cord blood HSCs. (A) flow cytometric analysis of fresh CD34+ enriched cells. Specific staining was performed with anti-CD34 FITC and anti-CD38 PE antibodies. Flow cytometric analysis of apoptosis at day 14 in different culture condition of CD34+ with and without ADSCs feeder layer by PI and Annexin V staining. (B) cord blood CD34+ in co-culture with ADSCs, and (C) cord blood CD34+ without ADSCs feeder layer

**Figure 5:**
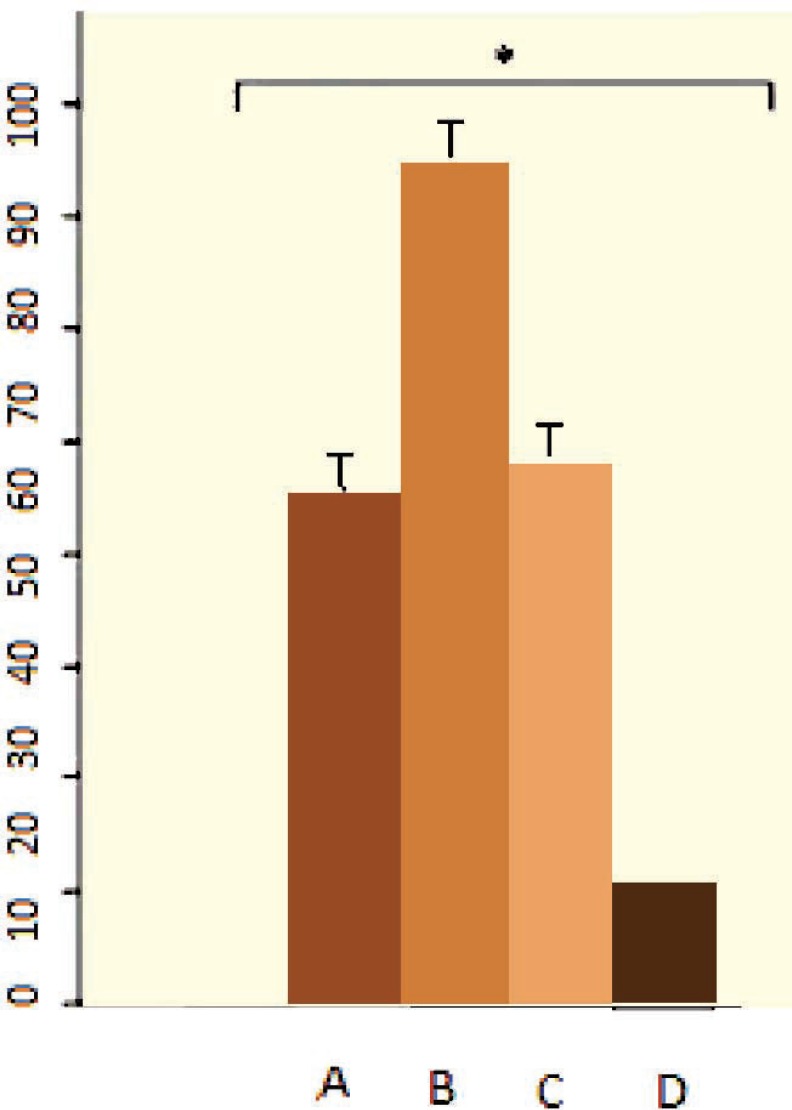
MTT analysis in different culture conditions. (A) CD34+ cells that indirectly cultured on feeder layer, (B) CD34+ cells directly cultured on a ADSCs feeder layer, (C) CD34+ cells in the presence of cytokines, and, (D) fresh CD34+ cells before culture

Differentiation potential of ASCs to adipocytes and osteoblasts was analyzed by Oil Red and Alizarin Red staining. Differentiated cells were positive for these staining (Fig 3). Separated CD34 + HSCs, analyzed by flow cytometry, were 88.8%; 22.2% of them were positive for CD38. *Ex vivo* expansion of HCB enriched CD34+ cells in serum-free medium supplemented with SCF, TPO and FLT3L, was evaluated either with or without feeder layer using flow cytometry (Fig 4). Annexin V and PI staining was performed for apoptosis analysis of expanded cells cultured. The percentages of apoptotic cells for PI and Annexin in co-culture with and without feeder layer are shown in Figure 4. The data of MTT assay showed that the proliferation rate of CD34+ cells directly cultured on a ADSCs feeder layer group was higher than the other group (Fig 5).

**Figure 6:**
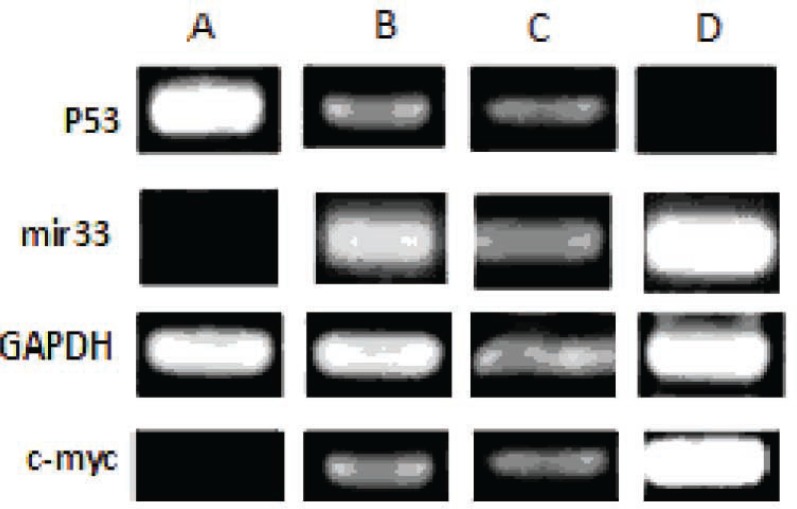
Results of RT-PCR. (A) CD34+ cells (the control group). In this group expression of *p53* was higher and *miR-33a* expression was lower than those of other groups. (B) CD34+ cells that only cultured with cytokines, (C) CD34+ cells cultured indirectly on a feeder layer, (D) CD34+ cells directly cultured on an ADSCs feeder layer. In this group, expression of *miR-33a* was higher than that in other groups

**Figure 7:**
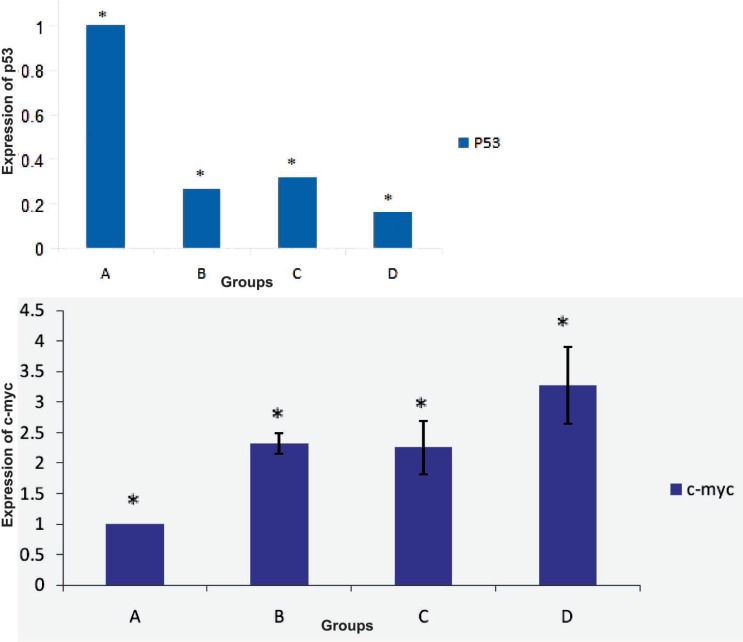
A) analysis of *p53* and *c-myc* genes expression in fresh CD34+ cells by RT-PCR. In this group, expression of *p53* was higher and that of *c-myc* was lower than those in other groups. (B) expression of *p53* and *c-myc* in CD34+ cells in the presence of cytokines. (C) expression of *p53* and *c-myc* in CD34+ cells that indirectly cultured on the feeder layer; expression of *p53* in groups B and C was similar. (D) expression of *p53* and *c-myc* in CD34+ cells that directly cultured on the feeder layer; in this group, expression of *p53* was lower than that in other groups (p<0.05

Results of RT-PCR showed that the expression of *miR-33a* and *c-myc* in HSCs directly cultured on ASCs feeder layer group was higher than those of other groups. Also the expression level of *p53* in this group was lower than those of other groups (Fig 5). Results showed that *miR-33a* and *p53* negatively regulated each other. RT-PCR analysis showed expression of *miR-33a* in groups where HSCs indirectly cultured on feeder layer and groups where HSCs cultured in the presence of the above-mentioned cytokines (Fig 6). Our results showed that the expressions of *miR-33a* and *p53* genes in ThinCert™ Plate with a pore size of 0.4 µm were lower than those of HSCs cultured directly on ASCs feeder layer. The direct contact between HSCs and feeder layer was prevented by a microporous membrane; consequently, the expression of *p53* in this group increased compared to that expressed when there was a direct contact of the feeder layer with hHSCs (Fig 7).

**Figure 8:**
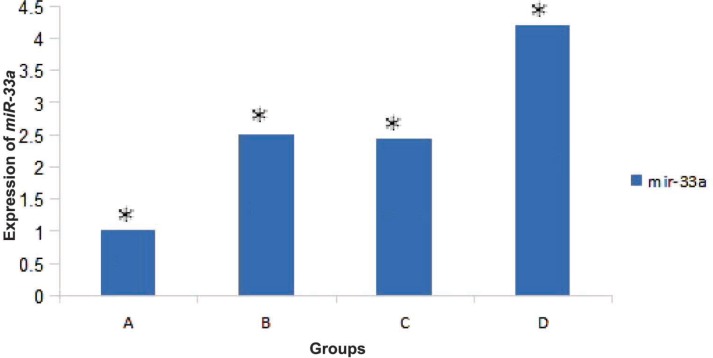
A) Analysis of *miR-33a* expression in fresh CD34+ cells by RT-PCR. (B) Expression of *mirR-33a* in CD34+ cells in the presence of cytokines. (C) Expression of *miR-33a* in CD34+ cells that indirectly cultured on the feeder layer; expression of *miR-33a* in groups B and C was similar. (D) expression of *miR-33a* in CD34+ cells that directly cultured on the feeder layer; in this group expression of *miR-33a* was higher than that in other groups (p<0.05

Results of RT-PCR analysis showed that expression of *miR-33a* in groups where HSCs were directly cultured on the feeder layer was significantly (p<0.05) higher than that in other groups (Fig 8).

In the control group, the expression of *miR-33a* was lower because HSCs in this group was quiescent. The highest expression level of *p53* gene was observed in CD34+ hHSCs (p<0.05). Expression of *p53* in the presence of the feeder layer was lower than that in other experiments (p<0.05).

## DISCUSSION

Our results showed that self-renewal of HSCs was higher in the presence of ADSCs feeder layer compared with other groups. Because of insufficient number of HSCs, the expansion of these cells is important for clinical application. Recent studies reported that bone marrow mesenchymal stem cells, as a feeder layer, and cytokines such as SCF and TPO increase proliferation of HSCs [2, 19]. Glettig, *et al*, showed that different feeder layers for HSCs limit the differentiation of these cells [20]. Our data revealed that expression of *p53*, as a self-renewal inhibitor gene, in HSCs cultured on feeder layer was lower than that in other groups. Our results showed that expressions of *miR-33a* and *p53* genes in ThinCert™ Plate with a pore size of 0.4 µm were lower than those in HSCs cultured directly on ASCs feeder layer. It has been reported that direct contact between HSCs and the feeder layer was critical for expansion of cells. Silva, *et al*, reported that direct contact of HSCs and feeder layer can increase self-renewal of HSCs [21]. Alakel, *et al*, showed that direct contact between HSCs and bone marrow mesenchymal stem cells feeder layer could improve the self-renewal of HSCs and can affect the migratory behavior of HSCs [22]. Based on the results of the present study, HSCs directly cultured on ADSCs presented higher levels of *c-myc* and *miR-33a* expressions than those in groups with indirect contact (ThinCert™ Plate). These results showed that high expression of *miR-33a* in the presence of ASCs feeder layer could down-regulate the *p53* and enhance the expansion of HSCs. We also showed that the expression level of *c-myc* was higher in HSCs cultured on the feeder layer compared with those cultured without ADSCs feeder. In contrast, it was shown that *c-myc* was negatively regulated by *miR-33* at the post-transcriptional level, via a specific target site within the 3’UTR and over-expression of *c-myc* impaired miR-33b-induced inhibition of proliferation and invasion in osteosarcoma cells [16]. It seems that *miR-33* mediated down-regulation of *p53*. Xu, *et al*, found that *miR-33* Inhibits tumoral cell migration and invasion by targeting the *c-myc* gene, suppreses tumors. On the other hand, another research showed that *miR-33* reduces cell proliferation and cell cycle progression and impairing the *p53* tumor suppressor gene function [23]. Perhaps, it explains that the expression level of *miR-33* depends on the cell type. The findings of this study contributed to our understanding of the function of *miR-33a* in HSCs cultured on ADSCs, as a down-regulator of *p53*. Although various reports indicated that *miR-33* inhibits tumoral cell migration and invasion by targeting the *c-myc* gene, acting as a tumor suppressor. It seems that factors secreted by adipose stem cells in the feeder layer targeted mir-33-mediated down-regulation of *p53* in expansion of HSCs.

In conclusion, it seems that *miR-33* increases proliferation of HSCs cultured on ADSCs by impairing the *p53* function. Defining the role of ADSCs in controlling the HSCs self-renewal through increased *miR-33* and reduced *p53* may lead to the treatment and prevention of hematopoietic disorders. Improvement in self-renewal of HSCs directly cultured on ADSCs was associated with increased expression of *miR-33* and *c-myc* and decreased expression of *p53*.
